# Combined radioligand therapy and immunotherapy for cancer: research advances and translational challenges

**DOI:** 10.3389/fphar.2026.1949065

**Published:** 2026-09-14

**Authors:** Sen Wang, Shoujun Tang, Shengjie Tang, Chun Wang, Junjie Wang, Yunlong Yang, Yang Yang, Chengkuan Liu, Jiang Fu, Haining Zhou

**Affiliations:** 1 Clinical Medical College, North Sichuan Medical College, Nanchong, China; 2 Department of Thoracic Surgery, Suining Central Hospital, Suining, China

**Keywords:** clinical translation, immune checkpoint inhibitors, immunotherapy, radioligand therapy, tumor immune microenvironment

## Abstract

Radioligand therapy (RLT) delivers therapeutic radionuclides to tumor-associated targets through specific targeting ligands and has demonstrated substantial clinical value in malignancies such as prostate cancer and neuroendocrine tumors. Beyond its direct induction of radiation-mediated tumor cell damage, RLT has attracted increasing attention for its potential immunomodulatory effects. Current evidence suggests that RLT may create local immune conditions favorable for immunotherapy by promoting tumor antigen release, enhancing antigen presentation, and modulating the tumor immune microenvironment. Immunotherapy, particularly immune checkpoint inhibitors, can restore or enhance T cell-mediated antitumor immune responses. Accordingly, RLT and immunotherapy are mechanistically complementary and have shown synergistic antitumor activity in several preclinical models. However, clinical evidence supporting this combination remains preliminary, and whether preclinical synergy can be consistently translated into meaningful clinical benefit is still unclear. Following a “mechanism–evidence–translation” framework, this review summarizes the radioimmunological rationale, preclinical evidence, and clinical progress of RLT combined with immunotherapy. It also provides a descriptive overview of the available evidence and discusses key translational challenges, including dosimetry, treatment sequencing, patient selection, and long-term safety, with the aim of informing the optimization of future combination strategies and clinical trial design.

## 1 Introduction

In recent years, rapid advances in nuclear medicine, molecular imaging, and radiopharmaceutical research have established radionuclide therapy as an increasingly important component of precision oncology (Sgouros et al., 2020; Zhang S. et al., 2025). Unlike conventional external beam radiotherapy (EBRT), radiopharmaceutical therapy delivers therapeutic radionuclides to tumor-associated targets through specific targeting vectors, thereby inducing localized radiation-mediated cytotoxicity while addressing systemic disease. This approach provides a therapeutic option for patients with advanced, metastatic, or refractory malignancies (Zhang S. et al., 2025; Goldsmith, 2020). Among these strategies, radioligand therapy (RLT) couples therapeutic radionuclides to small molecules, peptides, or other targeting ligands, enabling selective recognition and precise delivery to tumor cells or tumor-associated targets. RLT has demonstrated substantial clinical value in prostate cancer, neuroendocrine tumors, and other malignancies (Ninatti et al., 2025; To et al., 2025; Sartor et al., 2021; Singh et al., 2024).

As research on RLT has advanced, its effects are no longer considered limited to the direct radiation-mediated killing of tumor cells. Current studies of RLT and related radiopharmaceutical therapies suggest that RLT-induced DNA damage and tumor cell death may be accompanied by tumor antigen release, enhanced antigen presentation, and remodeling of the tumor immune microenvironment, thereby influencing host antitumor immune responses. These features indicate that RLT is not only a therapeutic modality for the precise delivery of radionuclides but may also contribute to the regulation of tumor immune status under certain conditions (Shea et al., 2024; Sun et al., 2023). Because immunotherapy primarily acts by activating or restoring host antitumor immune responses, it may be mechanistically complementary to RLT. Accordingly, combination strategies integrating RLT with immunotherapy have attracted increasing attention (Rui et al., 2023; Bellavia et al., 2022; Jiao and Dadachova, 2025).

To date, the combination of RLT and immunotherapy has shown synergistic antitumor potential in several preclinical models and has gradually entered early clinical exploration. However, its actual clinical benefit, suitable patient populations, and optimized application strategies remain to be clarified (Bellavia et al., 2022; Jiao and Dadachova, 2025; Kerr et al., 2022). Based on these considerations, this review focuses on RLT and summarizes the mechanistic rationale, preclinical evidence, current clinical progress, and translational challenges associated with its combination with immunotherapy. In addition, the available evidence is narratively synthesized and critically discussed to inform the optimization of future combination strategies and the design of clinical studies.

## 2 Overview of radioligand therapy and immunotherapy

### 2.1 Overview of RLT

RLT is a precision targeted therapeutic strategy for cancer. Its basic principle is to couple therapeutic radionuclides with ligands that specifically recognize tumor-associated receptors or antigens, thereby enabling selective radionuclide accumulation in tumor tissues and inducing DNA damage and tumor cell death through radiation-mediated cytotoxicity (Ninatti et al., 2025; Mittra et al., 2024). Compared with conventional external beam radiotherapy (EBRT), RLT is characterized by targeted delivery and systemic therapeutic activity. It can increase radiation exposure within tumors while potentially limiting exposure to selected non-target tissues, although uptake in dose-limiting organs remains clinically relevant (To et al., 2025). Notably, in nuclear medicine and oncology research, the concepts of RLT, targeted radionuclide therapy (TRT), and radiopharmaceutical therapy (RPT) partially overlap but are not entirely interchangeable. RPT refers to the broad category of therapeutic radiopharmaceuticals, TRT emphasizes the selective delivery of radionuclides to tumor-associated targets or lesions, whereas RLT is more commonly used to describe therapeutic approaches composed of targeting ligands and therapeutic radionuclides (Al-Ibraheem et al., 2025). Therefore, this review focuses on RLT and appropriately includes TRT/RPT studies with similar mechanisms and targeted delivery characteristics as supplementary evidence, while studies on EBRT are used only to explain relevant radioimmunological mechanisms.

#### 2.1.1 Common therapeutic radionuclides and their characteristics

According to radiation type, radionuclides commonly used in RLT can be broadly classified as β-particle emitters, α-particle emitters, and Auger electron emitters. These radionuclide classes differ markedly in linear energy transfer (LET), tissue penetration range, and biological effects; consequently, their suitable lesion characteristics and stages of clinical translation also vary (Morris et al., 2021; Karimi et al., 2026; Song and Sgouros, 2024). The basic classification and main physical characteristics of commonly used therapeutic radionuclides are summarized in Table 1. Among these, β-particle emitters have relatively mature clinical applications, a moderate tissue penetration range, and good clinical accessibility, and have been widely used in radiopharmaceutical therapy for thyroid cancer, neuroendocrine tumors, prostate cancer, and other malignancies (Karimi et al., 2026; Song and Sgouros, 2024; Pomykala et al., 2023). α-particle emitters are characterized by high LET and a short penetration range, showing considerable potential for micrometastases, residual lesions, and certain refractory tumors (Karimi et al., 2026; Song and Sgouros, 2024). Auger electron emitters have an extremely short range and are highly dependent on subcellular localization, particularly nuclear localization; therefore, their current application remains largely at the preclinical stage (Ku et al., 2019; Pirovano et al., 2021).

**TABLE 1:** Basic classification and characteristics of common therapeutic radionuclides.

Radionuclide category	Linear energy transfer (LET, keV/μm)	Tissue penetration range	Representative radionuclides (half-life)
β-particle emitters	Approximately 0.2	Approximately 0.05–12 mm	^177^Lu(6.65 days), ^90^Y(64.05 h), ^131^I(8.02 days)
α-particle emitters	Approximately 50–230	Approximately 28–100 μm	^225^Ac(9.92 days), ^211^At(7.22 h), ^213^Bi(45.61 min)
Auger electron emitters	Approximately 4–25	<1 μm	^111^In(2.80 days), ^125^I(59.40 days)

The linear energy transfer (LET) and tissue penetration ranges listed in the table represent general or commonly reported ranges in the literature rather than fixed values. Representative radionuclides are provided only for illustrative classification. Radionuclide classification, LET, and tissue penetration ranges were mainly summarized based on Refs. (Morris et al., 2021; Karimi et al., 2026; Song and Sgouros, 2024; Ku et al., 2019), whereas half-lives were obtained from the NNDC, NuDat 3.0 database.

#### 2.1.2 Current applications of radioligand therapy in cancer

RLT has relatively mature clinical applications in metastatic castration-resistant prostate cancer (mCRPC) and neuroendocrine tumors. Among these, prostate-specific membrane antigen (PSMA)-targeted RLT has become an important therapeutic option for patients with PSMA-positive mCRPC, while α-emitter-based therapies, such as ^225^Ac-PSMA, have shown exploratory value in selected patients with refractory or progressive disease (Alam et al., 2022; Giraudet et al., 2021; Fallah et al., 2023). Somatostatin receptor (SSTR)-targeted peptide receptor radionuclide therapy (PRRT), represented by ^177^Lu-DOTATATE, has also established a relatively mature clinical basis in SSTR-positive neuroendocrine tumors (Singh et al., 2024; Hope et al., 2020; Becx et al., 2022). With the development of novel targets and radioligands, RLT is being increasingly explored across a broader range of solid tumors. However, its wider implementation remains limited by target heterogeneity, insufficient individualized dosimetry, treatment resistance, and normal tissue toxicity (Ninatti et al., 2025; Currie and Bailey, 2025). Beyond direct radiation-mediated cytotoxicity, RLT may also contribute to antitumor immune responses by influencing tumor antigen release and the immune microenvironment, thereby providing a theoretical basis for its combination with immunotherapy (Shea et al., 2024; Sun et al., 2023).

### 2.2 Overview of immunotherapy

#### 2.2.1 Major types and current applications of immunotherapy

Major forms of cancer immunotherapy currently include immune checkpoint inhibitors (ICIs), adoptive cell therapy (ACT), cancer vaccines, cytokine therapy, and other treatment modalities (Zhang M. et al., 2025). Among these, ICIs are among the most widely used immunotherapeutic approaches in oncology and are supported by relatively robust clinical evidence. Representative classes include PD-1/PD-L1 inhibitors and CTLA-4 inhibitors, which restore or enhance T cell-mediated antitumor immune responses primarily by blocking inhibitory immune checkpoint signals. ICIs have become important treatment options for multiple solid tumors, including melanoma, non-small cell lung cancer, renal cell carcinoma, and urothelial carcinoma (Naimi et al., 2022; Kong et al., 2024; Chen et al., 2025). ACT enhances antitumor effects through the reinfusion of immune cells with antitumor activity after *ex vivo* expansion or genetic engineering (Zhang et al., 2023; Albarrán et al., 2024). Among ACT approaches, chimeric antigen receptor T cell therapy has achieved remarkable efficacy in certain hematologic malignancies; however, its application in solid tumors remains limited by antigen heterogeneity, insufficient tumor infiltration, and an immunosuppressive microenvironment (Albarrán et al., 2024). In addition, cancer vaccines and cytokine therapy may exert antitumor effects by inducing tumor antigen-specific immune responses or modulating immune cell function, but their clinical translation and scope of application remain limited (Rui et al., 2023; Zhang M. et al., 2025).

#### 2.2.2 Limitations of immunotherapy

Despite substantial advances in immunotherapy, several limitations remain. Although ICIs have shown encouraging efficacy in many cancers, responses are generally more frequent in immunologically “hot” tumors, which are characterized by abundant immune cell infiltration and active inflammatory responses. In contrast, immunologically “cold” tumors with limited immune cell infiltration often show restricted therapeutic responses. In addition, tumor heterogeneity and interindividual differences contribute to variable patient responses, while treatment resistance and immune-related adverse events (irAEs) remain major challenges (Zhang M. et al., 2025; Naimi et al., 2022). Although ACT has achieved remarkable efficacy in hematologic malignancies, its application in solid tumors remains constrained by antigen heterogeneity, on-target/off-tumor toxicity, and an immunosuppressive microenvironment (Albarrán et al., 2024). The clinical translation of cancer vaccines and cytokine therapy is also affected by antigen selection, individual immune variability, and inconsistent therapeutic efficacy (Rui et al., 2023; Zhang M. et al., 2025). Therefore, enhancing tumor immunogenicity, remodeling the immunosuppressive microenvironment, and expanding the population eligible for immunotherapy remain important issues in current immunotherapy research. Because RLT can induce tumor cell damage, antigen release, and local immune modulation, it represents a potential strategy for combination with immunotherapy.

### 2.3 Potential mechanistic basis for combining RLT and immunotherapy

The rationale for combining RLT with immunotherapy lies in their potential mechanistic complementarity. Through the targeted delivery of therapeutic radionuclides, RLT can induce tumor cell DNA damage and death and may promote tumor antigen release, enhance antigen presentation, and modulate the tumor immune microenvironment (TIME) (Shea et al., 2024; Sun et al., 2023; Constanzo et al., 2023). Immunotherapy, particularly ICIs, can relieve T cell suppression and restore antitumor immune responses, thereby complementing RLT-induced immunogenic changes and microenvironmental remodeling (Bellavia et al., 2022; Kleinendorst et al., 2022; Pouget et al., 2023). Therefore, RLT may serve as a potential immunomodulatory strategy that creates a more favorable immune context for ICIs and other immunotherapeutic approaches. The potential mechanisms underlying this combination are illustrated in Figure 1.

**FIGURE 1:**
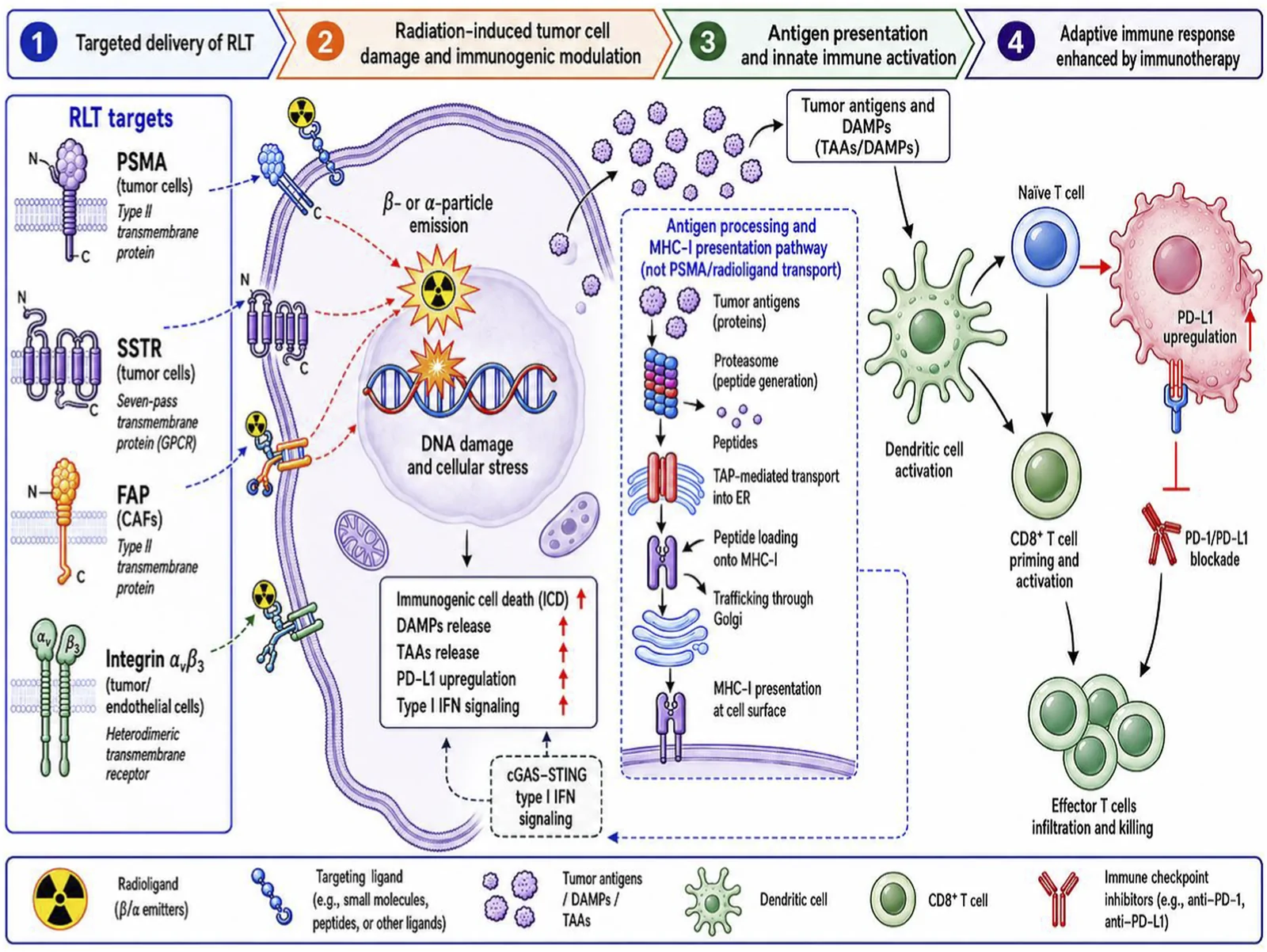
Potential immunological mechanisms underlying radioligand therapy (RLT) combined with immunotherapy. RLT targets tumor-associated molecules such as PSMA, SSTR, FAP and integrin αvβ3 to deliver therapeutic radionuclides. β- or α-particle emission induces DNA damage and immunogenic tumor-cell stress, promoting antigen release, DAMP exposure, including calreticulin exposure, dendritic-cell activation, antigen presentation and CD8^+^ T-cell priming. Radiation-induced cGAS–STING/type I IFN signaling and adaptive PD-L1 upregulation may further shape antitumor immunity, providing a mechanistic basis for combining RLT with immunotherapy, particularly immune checkpoint blockade. The ER/Golgi-related pathway shown in the figure represents antigen processing and MHC-I presentation rather than intracellular transport of PSMA or the radioligand.

Current understanding of radiation-induced antitumor immune responses is largely derived from studies of EBRT, which provide an important radioimmunological basis for understanding the combination of RLT and immunotherapy (Chen X. et al., 2025). However, RLT differs substantially from EBRT in irradiation mode, dose rate, and spatial dose distribution. Unlike EBRT, which delivers radiation from an external source without systemic administration of a pharmacological agent, RLT depends on the administration of a radiopharmaceutical and is therefore additionally influenced by target-dependent uptake, *in vivo* biodistribution, clearance, and heterogeneous radiation exposure in tumors and normal organs (Morris et al., 2021). Therefore, EBRT-related mechanisms should not be regarded as direct evidence for RLT. In the following sections, this review prioritizes evidence from RLT/TRT/RPT-related studies and uses EBRT studies only as theoretical support when direct evidence is insufficient.

#### 2.3.1 Immunogenic cell death and tumor antigen release

Beyond direct radiation-mediated tumor cell killing, RLT-induced cell death may also be immunogenic. Based on radioimmunological studies and emerging RLT/TRT-related evidence, ionizing radiation can induce immunogenic cell death (ICD) under certain conditions (Shea et al., 2024; Zhu et al., 2021; Gameiro et al., 2014). During this process, dying or stressed tumor cells may release tumor-associated antigens (TAAs) and display changes in damage-associated molecular patterns (DAMPs), including cell-surface exposure of calreticulin (CRT) and the release of adenosine triphosphate (ATP) and high mobility group box 1 (HMGB1). These signals can promote dendritic cell (DC) recruitment and maturation, as well as antigen uptake and cross-presentation, thereby supporting CD8^+^ T cell-mediated antigen-specific antitumor immune responses (Shea et al., 2024; Zhu et al., 2021; Vaes et al., 2021).

For therapeutic strategies that depend on pre-existing antitumor immunity, such as ICIs, insufficient tumor antigen release, inefficient antigen presentation, and limited immune recognition may compromise therapeutic efficacy (Mpakali and Stratikos, 2021). By inducing tumor cell damage and antigen release, RLT may enhance tumor immunogenicity and, to some extent, improve immune recognition of tumor cells. However, direct evidence supporting RLT-induced ICD remains more limited than that for EBRT, and its effects may be influenced by radionuclide type, tumor absorbed dose, dose rate, tumor biological characteristics, and the status of the immune microenvironment (Shea et al., 2024). Therefore, RLT-induced ICD and its role in combination immunotherapy require further validation in additional RLT/TRT/RPT-related studies.

#### 2.3.2 Enhanced antigen presentation and T cell activation

After tumor antigens are released, they must be taken up, processed, and presented by antigen-presenting cells (APCs) to effectively initiate adaptive antitumor immune responses. Among APCs, DCs play a central role in tumor antigen cross-presentation and T cell priming (Mpakali and Stratikos, 2021; Moussion and Delamarre, 2024). RLT-induced tumor cell death may increase the availability of tumor antigens for APCs, while the accompanying release or exposure of DAMPs can promote DC recruitment and maturation. Mature DCs can process internalized tumor antigens and present them to T cells through major histocompatibility complex (MHC) molecules. In particular, DCs can activate CD8^+^ T cells through MHC-I-mediated cross-presentation, thereby promoting the expansion and effector functions of cytotoxic T lymphocytes (CTLs) (Moussion and Delamarre, 2024).

“Cold tumors” are typically characterized by insufficient tumor antigen presentation, limited effector T cell infiltration, and enhanced immunosuppressive signaling, all of which restrict the efficacy of therapeutic strategies that rely on T cell-mediated antitumor immune responses (Liu et al., 2025). In this context, RLT may increase the immune visibility of tumor cells to some extent by inducing tumor cell damage, promoting antigen release, and enhancing antigen presentation, thereby facilitating CD8^+^ T cell-mediated recognition and killing of tumor cells.

#### 2.3.3 Remodeling of the tumor immune microenvironment

TIME is a key determinant of immunotherapy efficacy (Binnewies et al., 2018). Many solid tumors can develop an immunosuppressive TIME characterized by insufficient effector T cell infiltration, impaired antigen-presenting function of DCs, enrichment of regulatory T cells (Tregs) and myeloid-derived suppressor cells (MDSCs), and polarization of tumor-associated macrophages (TAMs) toward an M2-like or protumor immunosuppressive phenotype. Together, these changes can suppress CTL activation, expansion, tumor infiltration, and cytotoxic effector function, thereby limiting the efficacy of immunotherapeutic strategies such as ICIs (Tie et al., 2022).

In addition to promoting antigen release and antigen presentation, RLT may also influence antitumor immune responses by modulating TIME. Current RLT/RPT-related studies suggest that antitumor immune components, including CD8^+^ T cells, natural killer cells, and antigen-presenting cells, may increase in abundance or become activated after treatment, whereas immunosuppressive cells, such as Tregs, MDSCs, and TAMs, may also undergo changes in number or functional status (Shea et al., 2024; Sun et al., 2023). These changes suggest that RLT may, to some extent, alleviate local immunosuppression, thereby providing a microenvironmental basis for combination immunotherapy.

However, the effects of RLT on TIME are highly context-dependent and may be influenced by multiple factors, including radionuclide type, dosing regimen, tumor-targeting distribution, tumor biological characteristics, and baseline immune status (Shea et al., 2024; Morris et al., 2021). Therefore, the TIME-remodeling effects of RLT and the conditions under which RLT synergizes with immunotherapy require further clarification.

#### 2.3.4 DNA damage-mediated activation of innate immune pathways

Beyond adaptive immune responses, RLT-induced DNA damage may also contribute to antitumor immune modulation through innate immune pathways, such as cyclic GMP–AMP synthase–stimulator of interferon genes (cGAS–STING)/type I interferon (IFN) signaling. EBRT-related studies have shown that cytosolic DNA generated by radiation-induced damage can activate the cGAS–STING pathway, induce type I IFN responses, and promote DC function and effector T cell recruitment (Deng et al., 2014; Storozynsky and Hitt, 2020). Related RLT/TRT studies have also shown that ^90^Y-NM600 can induce STING-dependent type I IFN responses and enhance the antitumor effects of combination therapy with immune checkpoint blockade (Jagodinsky et al., 2021). However, given the differences between RLT/TRT and EBRT in dose rate, irradiation duration, and spatial dose distribution, the role of this pathway in different RLT regimens requires further clarification (Morris et al., 2021; Jagodinsky et al., 2021).

#### 2.3.5 Influence of radionuclide and target selection on immunomodulatory effects

The efficacy and immunomodulatory effects of RLT are not determined by administered activity alone, but are also influenced by absorbed dose, dose rate, radionuclide properties, and target biology. β-particle emitters, such as ^177^Lu and ^90^Y, have relatively low LET and a longer tissue penetration range, which may generate cross-fire irradiation and partly compensate for heterogeneous target expression, but may also affect adjacent immune cells or dose-limiting normal tissues (Morris et al., 2021; Karimi et al., 2026; Song and Sgouros, 2024). In contrast, α-particle emitters, such as ^225^Ac, deliver high-LET radiation over a short path length and can induce dense DNA damage in target-expressing cells, making their effects more dependent on target accessibility, microscopic radioligand distribution, and intratumoral uptake (Karimi et al., 2026; Song and Sgouros, 2024). Auger electron emitters require close proximity to critical subcellular structures, particularly DNA, and their immunomodulatory relevance remains less well established (Ku et al., 2019; Pirovano et al., 2021). Target selection may further influence immune consequences: tumor-cell-associated targets such as PSMA or SSTR may mainly promote tumor-cell damage and antigen release, whereas stromal or microenvironment-associated targets such as FAP may reshape the tumor stroma and immunosuppressive niche. Integrin αvβ3-targeted strategies may involve both tumor and angiogenesis-related compartments. Therefore, target expression, internalization, spatial distribution, tumor-to-normal uptake ratio, and lesion heterogeneity should be considered when interpreting the efficacy and immune effects of RLT combined with immunotherapy (Czernin et al., 2021; Chen et al., 2019; Chen J. et al., 2025; Zhao et al., 2024).

## 3 Preclinical evidence

### 3.1 RLT plus PD-1/PD-L1 inhibitors

PD-1/PD-L1 inhibitors are among the most commonly investigated combination partners in preclinical studies of RLT combined with immunotherapy. Overall, current evidence suggests that combining RLT with PD-1/PD-L1 blockade can enhance antitumor effects in selected tumor models. However, this efficacy is not universally observed, indicating that the therapeutic benefit of this strategy may be model-dependent.

For PD-1 blockade, PSMA-targeted RLT represents a relatively well-studied research direction. In a mouse model of prostate cancer, Czernin et al. found that combination therapy with ^225^Ac-PSMA-617 and an anti-PD-1 antibody further delayed tumor progression and prolonged survival compared with either monotherapy, suggesting a potential synergistic interaction between PSMA-targeted α-particle therapy and PD-1 blockade (Czernin et al., 2021).

Studies involving PD-L1 blockade have also shown synergistic potential. Using integrin αvβ3-targeted ^177^Lu-EB-RGD in an MC38 colon cancer mouse model, Chen et al. demonstrated that RLT/TRT combined with anti-PD-L1 therapy further inhibited tumor growth, prolonged survival, and enhanced protection against tumor rechallenge compared with either monotherapy. In addition, treatment sequencing influenced combination efficacy, with concurrent treatment showing superiority over delayed sequential therapy (Chen et al., 2019). Furthermore, FAP-targeted RLT combined with PD-L1 blockade has shown marked antitumor effects in multiple mouse tumor models. Mechanistic studies suggest that FAP-targeted radioligands can induce DNA damage, upregulate PD-L1 expression in tumor cells, enhance CD8^+^ T cell activation after combination with anti-PD-L1 therapy, and promote TIME remodeling. Complete responses and protection against tumor rechallenge were also observed in some models, suggesting that this strategy may induce protective antitumor immune memory (Chen J. et al., 2025; Zhao et al., 2024).

However, RLT combined with PD-1/PD-L1 inhibitors does not produce clear synergistic effects in all models. In a mouse model of prostate cancer, Potluri et al. found that ^90^Y-NM600 TRT increased intratumoral CD8^+^ T cell infiltration and altered the expression of immune checkpoint-related molecules, but its combination with anti-PD-1 therapy did not significantly improve antitumor efficacy. Further investigation suggested that anti-PD-1 therapy may enhance the suppressive activity of Tregs, thereby limiting combination efficacy; a more pronounced therapeutic improvement was observed only after Treg depletion (Potluri et al., 2022). These findings indicate that RLT-induced immune activation may be accompanied by immunosuppressive feedback, and whether PD-1/PD-L1 blockade provides additional benefit may depend on the specific tumor model and the status of the TIME.

### 3.2 RLT plus CTLA-4 inhibitors

Progress has also been made in preclinical studies combining RLT with CTLA-4 inhibitors. In an NF9006 breast cancer mouse model, Guzik et al. found that low-dose ^177^Lu-DOTA-folate combined with a CTLA-4 inhibitor suppressed tumor growth and prolonged survival more effectively than either monotherapy. Complete tumor regression was observed in some mice, with no apparent short-term adverse effects (Guzik et al., 2021). Rouanet et al. evaluated [^131^I]ICF01012-mediated melanin-targeted RLT combined with ICIs in a syngeneic B16F10 melanoma mouse model. The results showed that [^131^I]ICF01012 combined with a CTLA-4 inhibitor-containing regimen further prolonged survival compared with monotherapy, whereas the additional benefit of combining RLT with PD-1/PD-L1 blockade was relatively limited. These findings suggest that the synergistic effects between RLT and different immune checkpoint blockade strategies may vary (Rouanet et al., 2020).

In addition, Choi et al. evaluated VLA-4-targeted ^177^Lu-DOTA-PEG4-LLP2A (^177^Lu-LLP2A) as monotherapy and in combination with ICIs in a B16F10 melanoma mouse model. The study showed that ^177^Lu-LLP2A monotherapy achieved efficacy comparable to that of a dual-ICI regimen, whereas ^177^Lu-LLP2A combined with a CTLA-4 inhibitor-containing dual immune checkpoint blockade regimen further prolonged survival in mice (Choi et al., 2018). Notably, this study used TRT combined with dual ICIs rather than CTLA-4 inhibitor monotherapy as the combination partner; therefore, the independent contribution of CTLA-4 inhibition to this regimen requires further validation.

### 3.3 RLT combined with other immunotherapeutic strategies

Compared with studies of RLT combined with ICIs, research on RLT combined with other immunotherapeutic strategies remains limited, with the most direct available evidence mainly involving CAR-T cell therapy. In a mouse model bearing Hs746T human gastric cancer, Yang et al. found that low-dose ^177^Lu-DOTATATE enhanced the antitumor activity of CAR-T cells after their accumulation within tumors. The study also suggested that higher-dose TRT may damage both tumor cells and CAR-T cells, thereby reducing the efficacy of the combination (Yang et al., 2024). Sodji et al. similarly observed in a human GD2-positive neuroblastoma mouse model that low-dose ^177^Lu-NM600 followed by GD2 TRAC-CAR T cell therapy improved tumor control and prolonged survival, whereas higher RPT doses resulted in a lower rate of sustained complete responses. These findings suggest that an appropriate dose window may exist for combining RLT/RPT with CAR-T cell therapy (Sodji et al., 2025).

Overall, current preclinical studies of RLT combined with immunotherapy have shown some synergistic antitumor potential. However, the evidence is still mainly derived from animal models and is substantially influenced by tumor model, radionuclide type, radioligand, dosing regimen, and immunotherapy type. Meanwhile, some studies have not observed clear synergistic effects, and safety assessments, particularly long-term safety evaluations, remain relatively insufficient. Therefore, preclinical findings require further validation in subsequent clinical studies (Kleinendorst et al., 2025).

## 4 Current clinical research and translational challenges

Clinical exploration of RLT combined with immunotherapy remains at an early stage. Published and registered studies have mainly focused on combinations of RLT or PRRT with ICIs, with PSMA-targeted RLT and SSTR-targeted PRRT representing the major research directions. Therefore, this section focuses on clinical studies of PSMA-targeted RLT and SSTR-targeted PRRT combined with ICIs. Representative published and registered clinical studies are summarized descriptively in Table 2.

**TABLE 2:** Representative published and registered clinical studies of RLT/PRRT combined with ICIs.

Study/clinical trial	Design/status and sample size	Regimen and population	Main finding or current status	Main limitation
Published clinical reports and studies				
Aggarwal et al., NCT03805594	Phase I open-label dose-expansion study, n = 43	Single-dose ^177^Lu-PSMA-617 followed by pembrolizumab maintenance; mCRPC	Objective responses and acceptable safety were reported	Nonrandomized early-phase study; no RLT- or pembrolizumab-only control arm
PRINCE; NCT03658447	Phase Ib/II single-arm study, n = 37	Multi-cycle ^177^Lu-PSMA-617 combined with pembrolizumab; mCRPC	PSA responses and radiographic responses were reported in a subset of patients	Single-arm design; cannot determine the incremental contribution of pembrolizumab
Kim et al., NCT03325816	Phase I open-label study, n = 9	^177^Lu-DOTATATE combined with nivolumab; pulmonary neuroendocrine tumors/neuroendocrine carcinomas	Generally tolerable, with responses or disease stabilization in some patients	Very small sample size and heterogeneous disease population
Aicher et al.	Case reports, n = 2	Low-dose, short-interval ^177^Lu-DOTATATE combined with pembrolizumab; metastatic MCC	Short-term radiographic responses were observed	Case-based evidence only; limited generalizability
Ferdinandus et al.	Single-case report, n = 1	^177^Lu-DOTATOC combined with ipilimumab/nivolumab; metastatic MCC	Marked radiographic response was reported	Single-patient evidence; delayed immune effects and individual factors cannot be excluded
Registered studies without mature published results				
NCT05766371	Recruiting phase II study	^177^Lu-PSMA-617 combined with pembrolizumab; mCRPC	No mature formal results reported	Indicates research direction only
NCT03457948	Active, not recruiting	Pembrolizumab combined with liver-directed therapy or PRRT; well-differentiated NET with liver metastases	No mature formal full-text results available	Cannot be used to determine efficacy or safety
NCT04525638	Unknown status	^177^Lu-DOTATATE combined with nivolumab; G3 well-differentiated NET or poorly differentiated NEC	No mature formal results reported	Registry status is uncertain and requires cautious interpretation
GoTHAM; NCT04261855	Active, not recruiting; LuTate arm closed to recruitment	Avelumab combined with ^177^Lu-DOTATATE or EBRT; metastatic MCC	No mature formal results reported	Combination strategy remains exploratory
iPRRT; NCT05583708	Recruiting phase II study	^177^Lu-DOTATATE combined with pembrolizumab; SSTR-positive MCC progressing after ICI therapy	No mature formal results reported	Indicates post-ICI progression research direction only

This table provides a descriptive summary of representative published and registered clinical studies and does not constitute an exhaustive systematic evidence map or formal evidence-grading exercise. Registered trials without mature published results indicate research directions only and should not be used to draw conclusions regarding efficacy or safety. Trial status was last verified in ClinicalTrials.gov on 23 July 2026.

### 4.1 PSMA-targeted RLT combined with PD-1 inhibitors: clinical exploration in mCRPC

As shown in Table 2, PSMA-targeted RLT combined with PD-1 inhibitors is among the most clinically explored directions in current research on RLT combined with ICIs. In a phase I open-label dose-expansion study, Aggarwal et al. administered a single dose of ^177^Lu-PSMA-617 followed by pembrolizumab maintenance therapy and observed objective responses with acceptable safety in patients with mCRPC (Aggarwal et al., 2023). The PRINCE study conducted by Sandhu et al. (NCT03658447) further evaluated a multi-cycle ^177^Lu-PSMA-617 plus pembrolizumab regimen and reported a relatively high proportion of PSA responses, along with radiographic responses in some patients (Sandhu et al., 2026). These findings suggest that PSMA-targeted RLT combined with PD-1 inhibitors has preliminary clinical activity and feasibility.

However, these studies were early-phase, nonrandomized, or single-arm studies and lacked a ^177^Lu-PSMA-617 monotherapy arm or a pembrolizumab monotherapy arm as direct controls. Therefore, it remains difficult to determine whether the observed efficacy was mainly attributable to RLT itself, PD-1 blockade, or an additional synergistic benefit between the two modalities. In addition, differences across studies in RLT dosing cycles, immunotherapy maintenance strategies, prior treatment backgrounds, and PSMA expression levels limit direct comparison and generalizability of the results. Ongoing follow-up studies may help further evaluate the efficacy and safety of multi-cycle combination regimens; however, higher-quality controlled studies are still needed to validate the additional benefit of combination therapy.

### 4.2 SSTR-targeted PRRT combined with ICIs: exploration in neuroendocrine tumors and Merkel cell carcinoma

In addition to PSMA-targeted RLT, SSTR-targeted PRRT combined with ICIs represents another important direction in the clinical exploration of RLT combined with immunotherapy; however, its clinical evidence base is more limited than that for PSMA-targeted RLT combined with PD-1 inhibitors. A phase I study conducted by Kim et al. showed that ^177^Lu-DOTATATE combined with nivolumab was generally well tolerated in patients with pulmonary neuroendocrine tumors/neuroendocrine carcinomas, with responses or disease stabilization observed in some patients (Kim et al., 2020). However, the sample size was small, and the enrolled population included patients with small cell lung cancer, pulmonary carcinoids, and high-grade pulmonary neuroendocrine carcinomas, indicating substantial disease heterogeneity. Therefore, these findings mainly support further investigation, and it remains unclear whether ^177^Lu-DOTATATE combined with nivolumab is superior to PRRT monotherapy or PD-1 inhibitor monotherapy.

Merkel cell carcinoma (MCC) is a cutaneous malignancy with neuroendocrine features, and some lesions can express SSTR; therefore, SSTR-targeted PRRT has also been explored in immunotherapy-resistant or refractory MCC (Sergi et al., 2023; Askari et al., 2023). Existing case reports suggest that patients with SSTR-positive disease who progress after immunotherapy may derive short-term response signals from PRRT combined with ICIs (Aicher et al., 2022; Ferdinandus et al., 2022). However, this evidence is mainly derived from single-case reports, with complex patient backgrounds, substantial differences in prior treatments and immune status, and limited response duration. Therefore, the role of PRRT combined with ICIs in MCC should still be regarded as an exploratory salvage-treatment signal rather than as generalizable evidence of efficacy.

At present, several registered studies continue to explore SSTR-targeted PRRT combined with ICIs in NETs, NECs, and MCC. As summarized in Table 2, these studies mainly indicate ongoing research directions, and mature published results are still limited. Studies such as NCT03457948, NCT04525638, GoTHAM (NCT04261855), and iPRRT (NCT05583708) reflect the continued exploratory value of PRRT combined with ICIs in NETs, NECs, and MCC. However, before mature results are published, these registered studies can only indicate clinical research directions and cannot yet be used to determine efficacy or safety.

## 5 Discussion: translational challenges and optimization directions

As discussed above, RLT combined with immunotherapy has shown translational potential, but its clinical application remains at an early exploratory stage. In the future, a deeper understanding of the immunomodulatory mechanisms, dosimetric characteristics, treatment sequencing, and suitable patient populations for RLT will be needed to support regimen optimization and individualized application of combination strategies (Kerr et al., 2022). The main optimization directions, from mechanistic understanding to clinical translation, are shown in Figure 2.

**FIGURE 2:**
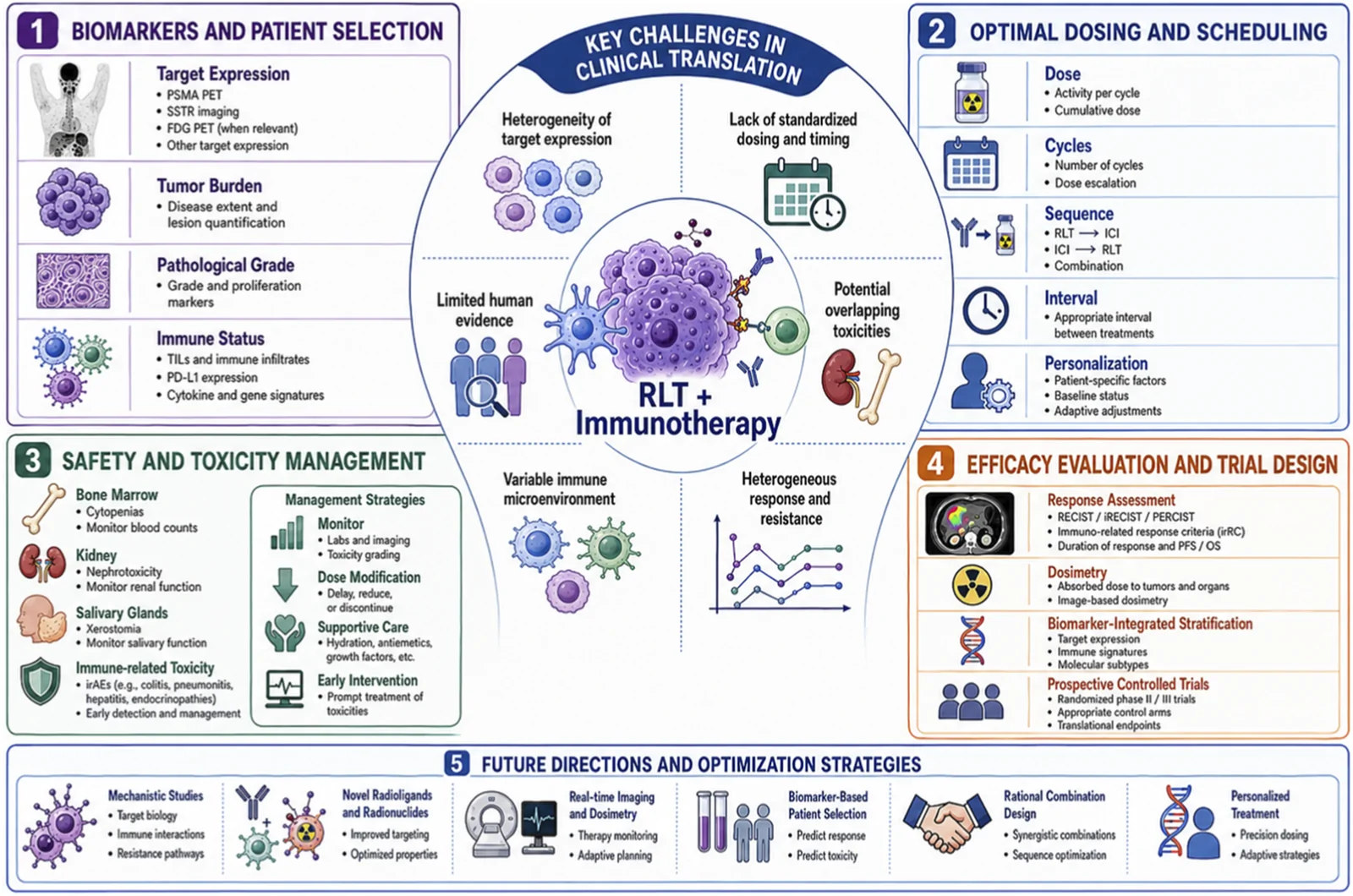
Clinical translation of radioligand therapy (RLT) combined with immunotherapy requires integrated optimization of patient selection, treatment sequencing, dosimetry, safety monitoring and response assessment. Biomarker-driven stratification, molecular imaging, individualized absorbed-dose evaluation and prospective clinical trials are needed to clarify additional clinical benefit and potential synergy, and to guide personalized application.

### 5.1 RLT-specific immunomodulatory mechanisms remain to be clarified

With the continued advancement of studies combining RLT with immunotherapy, the interaction between these two modalities has become an important scientific question for the future development of this field. Current understanding of radiation-induced antitumor immune responses is still largely derived from EBRT studies. Compared with EBRT, RLT has distinct radiobiological characteristics, including systemic administration, target-dependent uptake, heterogeneous spatial dose distribution, prolonged irradiation, and a relatively low dose rate; therefore, its effects cannot be simply inferred from EBRT-related mechanisms (Morris et al., 2021; Pouget et al., 2015). In this context, RLT-specific delivery characteristics may influence not only whether immune activation occurs, but also how strong and spatially coordinated that activation is (Morris et al., 2021; Constanzo et al., 2023; Pouget et al., 2023; Pouget et al., 2015). Prolonged low-dose-rate irradiation may generate a different temporal pattern of tumor-cell stress compared with the more controlled dose delivery of EBRT, whereas insufficient absorbed dose may fail to induce robust immunogenic modulation (Morris et al., 2021; Pouget et al., 2015; Bodei et al., 2024; Beauregard, 2022; O'Donoghue et al., 2022). In addition, heterogeneous radiopharmaceutical uptake among lesions and within individual tumors may lead to uneven immune priming, with highly irradiated regions becoming more immunogenic while poorly irradiated regions remain less affected or allow resistant subclones to persist (Bodei et al., 2024; Beauregard, 2022; O'Donoghue et al., 2022). Variable radiation exposure to stromal and immune-cell compartments may further modify the balance between antitumor immune activation and immunosuppressive feedback (Constanzo et al., 2023; Pouget et al., 2023). Therefore, RLT-induced immune effects should be interpreted as context-dependent outcomes shaped by target expression, biodistribution, absorbed dose, dose rate, spatial dose heterogeneity, and baseline immune status. Although combining RLT with immunotherapy has potential translational value, its clinical relevance depends on a deeper and continuing understanding of the interactions between these two modalities (Kerr et al., 2022; Pouget et al., 2023). Meanwhile, the integration of radiobiology, molecular imaging, and immunology may help move combination strategies from empirical use toward rational design with stronger biological foundations, thereby providing theoretical support for subsequent dose optimization, treatment sequencing, and individualized application.

### 5.2 Individualized dosimetry and assessment of immune effects

The efficacy and safety of RLT are closely related to the *in vivo* distribution of radiopharmaceuticals, tumor uptake, and normal tissue exposure. Because target expression and radiopharmaceutical uptake may vary substantially among patients and across lesions within the same patient, treatment intensity estimated solely from administered activity cannot adequately reflect actual radiation exposure (Bodei et al., 2024; Beauregard, 2022). With the increasing clinical use of RLT, dosimetry studies have gradually expanded from traditional assessments of organ toxicity to the interpretation of heterogeneous tumor responses and individualized treatment needs (Bodei et al., 2024). Although the relationship between absorbed dose and clinical benefit in RPT remains under active investigation (O'Donoghue et al., 2022), several studies of ^177^Lu-DOTATATE have suggested that absorbed doses to tumors or normal organs may be associated with lesion response, patient benefit, or toxicity risk (Hebert et al., 2024; Warfvinge et al., 2024). Therefore, further integrated analysis of absorbed dose, efficacy, safety, and immunotherapy response may help support the transition of RLT combined with immunotherapy from fixed-activity administration toward a more refined and individualized treatment evaluation model.

### 5.3 Optimization of treatment sequencing and combination regimens

Compared with monotherapy, RLT combined with immunotherapy involves multiple temporal dimensions, including the *in vivo* distribution and retention of radiopharmaceuticals, sustained radiation exposure, and cyclic administration of immunotherapy. Therefore, its efficacy may be influenced by administration sequence, treatment interval, number of RLT cycles, and duration of immunotherapy maintenance (Jiao and Dadachova, 2025; Chen et al., 2019; Aggarwal et al., 2023; Sandhu et al., 2026). Existing preclinical and early clinical studies suggest that different treatment sequences and combination regimens may affect both efficacy and safety. However, unified optimization criteria are still lacking. In the future, treatment sequencing and administration regimens should be further refined based on accumulating clinical evidence, while taking into account tumor type, radioligand characteristics, disease burden, and patient tolerability. This may help support the gradual development of more targeted and reproducible combination strategies.

### 5.4 Patient selection and individualized combination strategies

The clinical benefit of RLT usually depends on tumor target expression and radiopharmaceutical uptake, whereas the benefit of immunotherapy is associated with factors such as tumor immune status and patient clinical background (Ninatti et al., 2025; Doroshow et al., 2021; Havel et al., 2019). Therefore, combination strategies should place greater emphasis on selecting suitable patient populations and should involve comprehensive assessment of multiple factors, including molecular imaging, dosimetry, tumor burden, pathological grade, and immunological characteristics. For example, in PSMA-targeted RLT, PSMA PET and FDG PET have been used for pretreatment screening, response prediction, and prognostic evaluation in patients with mCRPC (Buteau et al., 2022). In SSTR-targeted PRRT, SSTR imaging provides an important basis for assessing target expression and selecting eligible patients, and relevant consensus recommendations emphasize that PRRT should be applied with comprehensive consideration of SSTR expression, tumor grade, disease progression, and organ function (Hope et al., 2020).

On this basis, future studies may further optimize patient selection and combination-regimen design by integrating imaging, pathological, and immunological characteristics, thereby promoting the development of RLT combined with immunotherapy toward more individualized and precision-based applications.

## 6 Conclusion

RLT combined with immunotherapy is based on the complementary effects of targeted radiation-mediated tumor killing and antitumor immune modulation, and has a clear theoretical rationale and translational potential. The targeted delivery of therapeutic radionuclides and the induction of radiation-mediated DNA damage and tumor-cell death represent the core biological basis of RLT. Building on this foundation, preclinical and early translational evidence suggests that RLT may, under certain conditions, promote tumor antigen release, enhance antigen presentation, activate innate immune pathways such as cGAS–STING/type I IFN signaling, and reshape the tumor immune microenvironment, thereby creating potentially favorable conditions for immunotherapy. However, the consistency and clinical relevance of these immunomodulatory effects remain to be further clarified across different radionuclides, molecular targets, tumor types, and immune backgrounds. Current preclinical studies and early clinical explorations generally suggest that this combination strategy may enhance antitumor effects in selected tumor types and under specific treatment conditions. Nevertheless, the true clinical benefit of combination therapy compared with monotherapy, as well as the suitable patient populations, has not yet been fully established.

Overall, RLT combined with immunotherapy provides a promising research direction for the treatment of advanced, metastatic, and refractory malignancies. Its clinical translation still faces several challenges, including incompletely defined RLT-specific immunomodulatory mechanisms, substantial heterogeneity in study design, nonuniform patient selection criteria, optimization of radionuclide and target selection, treatment sequencing and dosing regimens, and limited evidence regarding long-term safety. Future studies should rely on deeper mechanistic investigations and more rigorously designed prospective clinical trials to further define suitable patient populations, rational combination regimens, efficacy-predictive biomarkers, and long-term safety profiles across different radionuclides, targets, and immunotherapeutic strategies. These efforts may support the development of this strategy toward mechanism-driven, dose-optimized, and individualized precision therapy.
